# Closure of a secondary tracheoesophageal fistula in severe pneumonia using an Amplatzer Duct Occluder II during invasive mechanical ventilation: A case report

**DOI:** 10.1002/ccr3.9470

**Published:** 2024-10-16

**Authors:** Meng Fu, Dongsheng Wang, Jialiang Wang, Qixia Xu, Lejie Cao, Junqiang Zhang

**Affiliations:** ^1^ Department of Pulmonary and Critical Care Medicine, The First Affiliated Hospital of USTC, Division of Life Sciences and Medicine University of Science and Technology of China (USTC) Hefei Anhui China; ^2^ Science Island Branch, Graduate School of USTC University of Science and Technology of China (USTC) Hefei Anhui China; ^3^ Anhui Province Key Laboratory of Medical Physics and Technology, Institute of Health and Medical Technology, Hefei Institutes of Physical Science Chinese Academy of Sciences Hefei Anhui China; ^4^ Institute of Molecular Enzymology, School of Biology & Basic Medical Sciences Suzhou Medical College of Soochow University Suzhou Jiangsu China

**Keywords:** airway stenting, Amplatzer Duct Occluder II, bronchoscopy interventional treatment, efficacy, esophageal stenting, tracheoesophageal fistula

## Abstract

**Key Clinical Message:**

Early and timely closure of secondary tracheoesophageal fistula (TEF) is crucial for critically ill patients. For those requiring invasive mechanical ventilation, the Amplatzer Duct Occluder II (ADO II) can be used as an emergency therapeutic option to rapidly close secondary TEF, providing opportunities for subsequent treatments.

**Abstract:**

Secondary tracheoesophageal fistula (TEF) is a life‐threatening condition characterized by high mortality, high recurrence rates, and multiple complications. Reports on the management of secondary TEF in critically ill patients are limited due to the challenges in treatment and the lack of suitable therapeutic options. We report a case of secondary TEF in a 69‐year‐old male diagnosed with severe pneumonia, whose condition deteriorated rapidly following the onset of TEF. Despite invasive mechanical ventilation, maintaining blood oxygen saturation above 80% was unachievable due to the TEF. Bedside bronchoscopy revealed expansion TEF expansion caused by gastrointestinal fluid reflux and respiratory machine pressure. The TEF was urgently closed using an ADO II device during invasive mechanical ventilation to prevent further deterioration. After the patient's condition stabilized, the ADO II was replaced with a Y‐shaped tracheal membrane‐covered stent for further TEF management. The patient's condition improved, meeting the criteria for liberation from invasive mechanical ventilation, and bedside chest X‐rays revealed a gradual resolution of pulmonary inflammation. Selecting appropriate treatment modalities for early and timely closure of secondary TEF is crucial for critically ill patients. ADO II can serve as a rescue therapy to achieve rapid closure of secondary TEF in critically ill patients requiring invasive mechanical ventilation support, providing opportunities and time for subsequent treatment.

## 1 INTRODUCTION

Tracheoesophageal fistula (TEF) is an abnormal connection between the trachea and esophagus, broadly categorized into congenital and secondary types. Primary TEF is present from birth and results from a congenital abnormal connection between the trachea and esophagus. In contrast, secondary TEF is an acquired condition that develops after birth due to factors such as trauma, infection, or prolonged intubation. Secondary TEF is a life‐threatening condition characterized by high mortality, frequent recurrence, and multiple complications, which can be further classified into benign or malignancy‐related fistulae.^1^ Benign TEF is commonly associated with respiratory tract injuries, infectious diseases (such as tuberculosis, Crohn's disease, esophageal granulomatous diseases, syphilis, and fungal infections) or trauma, particularly following surgical procedures, placement of esophageal stents, or tracheal intubation.^2^, ^3^ Malignant TEF typically occurs as a complication of advanced esophageal carcinoma, advanced lung carcinoma, malignant tumors of the mediastinum, thyroid carcinoma, or metastatic tumors to the respiratory tract and following chest radiotherapy.^4^, ^5^, ^6^ However, reports on the management of secondary TEF in critically ill patients are extremely rare due to the challenging nature of treatment and the absence of suitable therapeutic modalities.^7^ Herein, we present a case of closing a secondary TEF in a patient with severe pneumonia, utilizing the ADO II during invasive mechanical ventilation.

## 2 CASE HISTORY/EXAMINATION

A 69‐year‐old man was admitted to our hospital on April 17, 2021, with severe pneumonia and difficulty weaning off invasive mechanical ventilation. He had been diagnosed with severe pneumonia 15 days earlier at a local hospital. His medical history included poorly differentiated squamous cell carcinoma of the mid‐esophagus, for which he underwent radical esophagectomy (left transthoracic esophagectomy and esophagogastrostomy above the aortic arch) in June 2005, followed by adjuvant chemotherapy and radiotherapy.

Upon admission to our emergency department, the patient was severely malnourished and dyspneic, with a body mass index (BMI) of 16.32 kg/m^2^. Physical examination revealed a respiratory rate (RR) of ≥ 30 breaths per minute, coarse breath sounds in both lungs with crepitations in the lower lobes, a heart rate of 104 beats per minute with a regular rhythm, and no murmurs. Pitting edema was present in both lower extremities. Blood gas analysis showed a pH of 7.31, a partial pressure of carbon dioxide (PaCO₂) of 75.6 mmHg, and a partial pressure of oxygen (PaO₂) of 66.3 mmHg, with an oxygenation index of 228.6 on 2 L/min of oxygen. Laboratory tests revealed a white blood cell count of 3.07 × 10^9^/L, hemoglobin of 80 g/L, and C‐reactive protein of 58.28 mg/dL. Biochemical analysis showed hypocalcemia (1.96 mmol/L), hypophosphatemia (0.65 mmol/L), and hypoproteinemia (32.3 g/L).

## 3 METHODS

Chest computed tomography (CT) demonstrated diffuse patchy opacities in both lungs accompanied by pleural effusion. Bedside bronchoscopy revealed abundant purulent discharge in the right middle and lower lobes, and bronchoalveolar lavage fluid (BALF) samples were collected for pathogen identification through traditional cultures and metagenomic next‐generation sequencing (mNGS). Aspiration of purulent discharge revealed yellow fluid overflowing from the bronchial lumen distal to the dorsal segment of the right lower lobe, which persisted despite lavage, suggesting infection‐related bronchopleural fistula. Based on positive findings for *Aspergillus* in both BALF culture and mNGS, antifungal therapy with voriconazole was initiated (loading dose of 300 mg every 12 h, followed by 200 mg every 12 h), tailored to the patient's weight (52 kg) and normal liver and kidney function.

**FIGURE 1:**
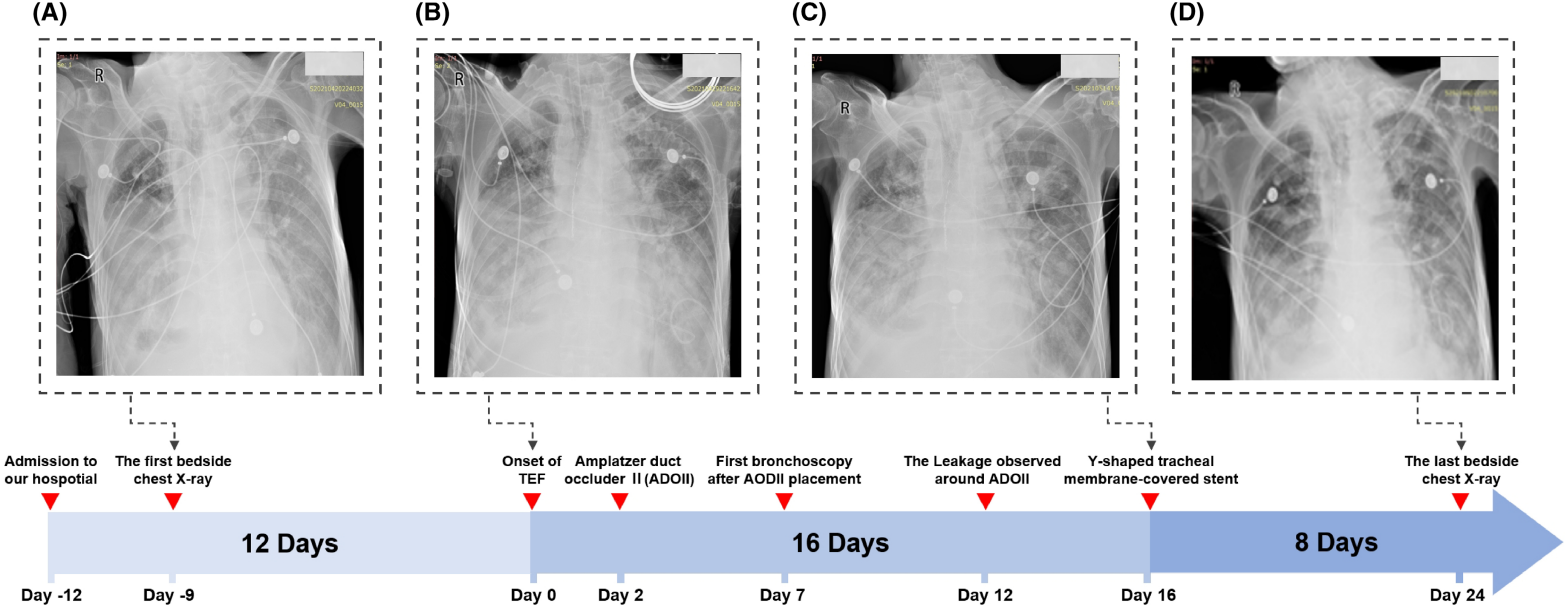
Timeline of chest imaging findings in a patient with tracheoesophageal fistula (TEF) treated with Amplatzer Duct Occluder II (ADO II) and tracheal stenting. (A) The first bedside chest X‐ray performed 12 days after admission shows diffuse bilateral pulmonary infiltrates, consistent with inflammatory exudates. (B) On the day of TEF onset, a chest X‐ray reveals predominant inflammatory infiltrates in the lower lung fields bilaterally. (C) Two weeks after ADO II placement, the bedside chest X‐ray demonstrates persistent pulmonary infiltrates without significant improvement. (D) Eight days after the insertion of a Y‐shaped covered tracheal stent, a bedside chest X‐ray shows a gradual reduction in pulmonary inflammation.

Additional management included thoracic drainage, fluid volume control, stabilization of the internal environment, and nutritional support. Nine days post‐admission, the initial bedside chest X‐ray revealed diffuse bilateral lung inflammation with exudates (Figure 1A). After 12 days of medical management, the patient's condition significantly improved, allowing for liberation from invasive mechanical ventilation and a transition to intermittent non‐invasive ventilation.

**FIGURE 2:**
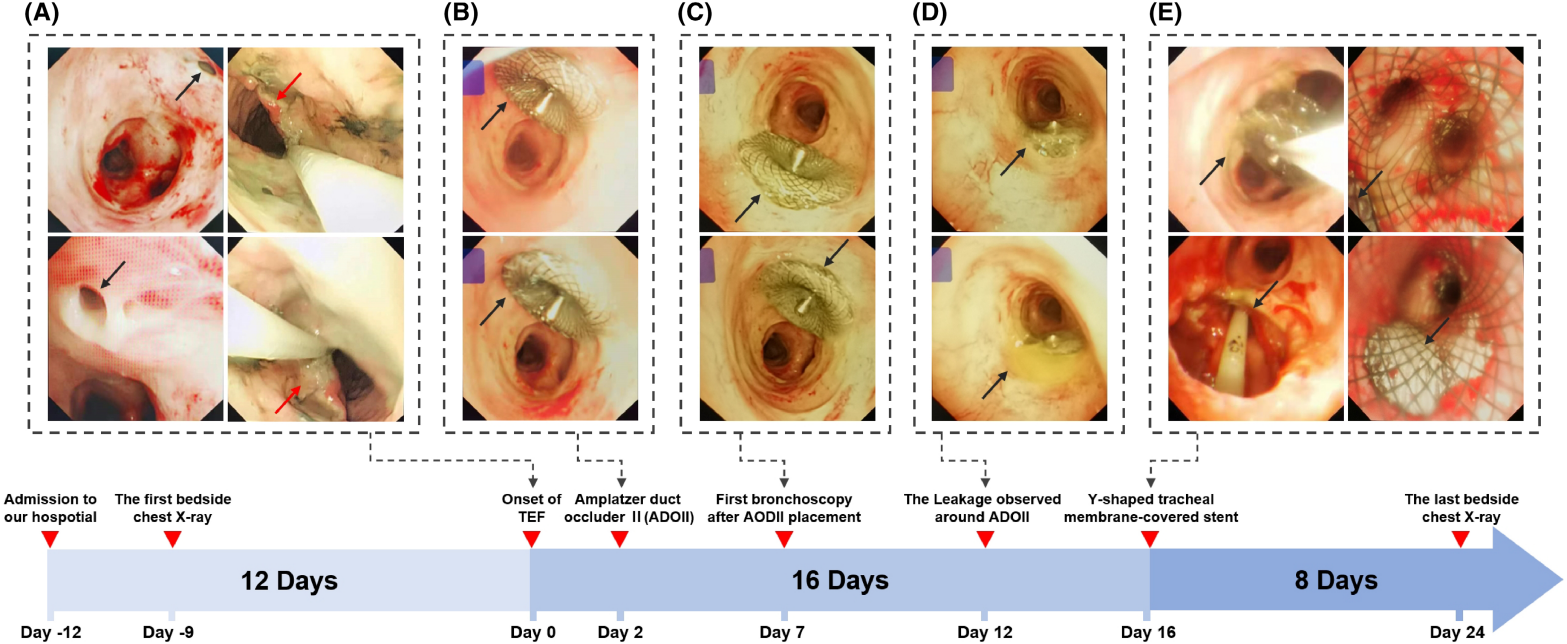
Timeline of treatment for tracheoesophageal fistula (TEF). (A) Initial bedside bronchoscopy identified a TEF measuring 8 mm in diameter (black arrow). Concurrent bedside gastroscopy revealed thickened, rigid, and erosive esophageal mucosa surrounding the TEF (red arrow), suggesting a probable recurrence of esophageal carcinoma. (B) Two days after the onset of TEF, an Amplatzer Duct Occluder II (ADO II) device was deployed to close the fistula during invasive mechanical ventilation. (C) Five days post‐ADO II placement, bedside bronchoscopy showed that the esophagotracheal fistula remained unhealed, with minor leakage observed around the occluder's edge. (D) Ten days following the placement of the ADO II, the fistula demonstrated an increased size, indicating progressive worsening. (E) Two weeks after the initial ADO II deployment, the device was replaced with a Y‐shaped tracheal membrane‐covered stent to achieve a more effective and stable closure of the TEF.

On the afternoon of April 29, 2021, the patient experienced a sudden deterioration in clinical condition, marked by severe carbon dioxide retention (pH 7.141, PaCO_2_>130mmHg). The primary cause was suspected to be airway obstruction due to the presence of dark liquid discharged from the patient's mouth. Emergency endotracheal intubation and invasive mechanical ventilation were initiated following extensive aspiration. Stabilization of vital signs was achieved, and bedside bronchoscopy under invasive ventilation revealed an 8 mm TEF (Figure 2A, black arrow). Bedside gastroscopy showed thickened, rigid, erosive esophageal mucosa surrounding the TEF, suggestive of recurrent esophageal cancer (Figure 2A, red arrow).

The bedside chest X‐ray following the patient's sudden deterioration revealed that the bilateral pulmonary exudates were not more severe than in the previous examination, indicating an acute progression of the patient's condition (Figure 1B). Due to the presence of TEF, even under invasive mechanical ventilation support, invasive mechanical ventilation was insufficient to maintain the patient's blood oxygen saturation level above 80%. Furthermore, bedside bronchoscopy examination indicates that the area of the TEF continued to expand due to gastrointestinal fluid reflux and respiratory machine pressure. Two days after the onset of the TEF, we opted for urgent closure of the TEF using an ADO II during invasive mechanical ventilation to prevent exacerbation of the condition (Figure 2B). In this procedure, a bedside bronchoscope was introduced through the patient's airway to visualize the TEF and determine its size and location. The Amplatzer Duct Occluder II (ADO II), preloaded in a delivery sheath, is advanced through the catheter. Under continuous fluoroscopic imaging, the device was precisely positioned across the TEF. The sheath is retracted to allow the ADO II to expand and occlude the fistula. Repeated observations with the bronchoscope confirmed the accurate placement of the ADO II at the site of the fistula.

Five days post‐ADO II placement, bedside bronchoscopy examination indicated that the esophagotracheal fistula had not healed, with minimal leakage around the occluder edge (Figure 2C). Ten days after the placement of ADO II, the fistula remained unhealed and showed an increasing trend, possibly due to TEF secondary to recurrent esophageal cancer (Figure 2D). Following stabilization of the patient's condition, we replaced the ADO II with a Y‐shaped tracheal membrane‐covered stent for further management of the TEF (Figure 2E). Under bedside bronchoscopy, a guidewire was introduced through the access site, and a retrieval catheter was advanced over the guidewire to the location of the ADO II. Using fluoroscopic guidance, the ADO II is carefully captured and withdrawn into the retrieval catheter. Fluoroscopy confirmed that the device is completely removed, with no residual fragments remaining. Following the successful removal of the ADO II, the catheter is replaced with one designed to deliver a Y‐shaped tracheal membrane‐covered stent. The stent, preloaded in the delivery sheath, was then advanced through the catheter to the TEF site. Under continuous fluoroscopic and bronchoscopic imaging, the stent was precisely positioned across the fistula. The sheath was retracted to deploy the stent, which expands to conform to the trachea and esophagus, effectively sealing the TEF. Repeated bronchoscopic observations confirmed the precise placement and adequate expansion of the Y‐shaped stent. Due to the superior occlusion effect of the Y‐shaped tracheal membrane‐covered stent on the TEF, the pressure levels required for the patient's invasive mechanical ventilation support gradually decreased. The bedside chest X‐ray showed that the diffuse pulmonary exudates in both lungs had improved compared to previous examinations (Figure 1C).

## 4 CONCLUSION AND RESULTS

The patient tolerated the Y‐shaped tracheal membrane‐covered stent well, demonstrating clinical improvement and meeting the criteria for liberation from invasive mechanical ventilation. Bedside chest X‐rays revealed gradual resolution of pulmonary inflammation (Figure 1D). Unfortunately, the patient's legal representative opted to discontinue treatment upon learning of the recurrence of esophageal cancer, as confirmed by pathological examination. Eventually, the patient succumbed to respiratory failure and pulmonary infection 3 days post‐discharge.

## 5 DISCUSSION

When the integrity of the respiratory tract wall is compromised for various reasons, the formation of a fistula in the wall is referred to as a respiratory tract fistula. If the fistula creates a connection between the lower respiratory tract and the digestive tract, allowing digestive contents to enter the respiratory tract, it may lead to pulmonary infection and respiratory failure; the severity of symptoms depends on the size of the fistula, the amount of regurgitation, and the primary illness.^8^ Malignant TEF typically occurs as a complication of advanced malignant thoracic tumors, with the majority being secondary to advanced esophageal carcinoma, which comprises over 70% of cases. Approximately 0.2%–5% of patients with esophageal carcinoma and 1% of patients with lung carcinoma may develop TEF.^4^, ^5^, ^8^, ^9^ For patients with benign secondary TEF, if surgical opportunities arise, efforts should be made to surgically remove the fistula and affected tissue.^10^ However, patients with malignant secondary TEF are generally in advanced stages of cancer, with poor physical condition, and have poor physical conditions, making most unsuitable for surgical treatment, often only able to undergo interventional therapy or conservative medical treatment.^11^ Interventional therapy under bronchoscopy, gastroscopy, and imaging guidance is the main treatment method for secondary TEF not suitable for surgery, greatly alleviating patients' symptoms and improve their quality of life. Currently, the predominant approach involves the placement of respiratory and/or digestive stents.^12^ The treatment of TEF typically involves various types of airway stents, which are primarily used to maintain airway patency and prevent the abnormal communication between the trachea and esophagus from affecting breathing and swallowing functions. The following are some common types of airway stents: (1) Metal stents: Usually made of nitinol or stainless steel, these stents offer excellent support and flexibility. They can expand and reinforce damaged airway walls while tolerating pressure changes within the airway; (2) Adjustable stents: Some modern stents have adjustable features, allowing for modifications according to the patient’s condition to fit different anatomical structures; (3) Silicone stents: These stents are often used for patients requiring long‐term support. The silicone material is biocompatible and easy to remove and replace; (4) Covered stents: These stents are coated with a thin membrane that prevents food or liquids from entering the airway from the esophagus, thereby reducing the risk of complications.

The European Society of Gastrointestinal Endoscopy (ESGE) Clinical Guideline recommends the use of partially or fully covered self‐expandable metal stents (SEMSs) as the preferred therapeutic approach for TEF associated with malignant disease.^13^ Since the 1990s, the primary method for closing fistulas in patients with malignant TEF undergoing stent placement has been the use of SEMSs in the esophagus. Results from multiple retrospective studies have shown that the success rate of fistula closure with SEMSs for treating TEF ranges from 70% to 100%.^14^ Clinical guidelines from the ESGE recommend the primary use of esophageal SEMSs for treating malignant TEF, although the optimal duration of stent placement remains unclear, and individualized treatment principles should be followed. Critically ill patients with severe acute dyspnea due to malignant tracheal stenosis or TEF often need advanced respiratory support.^15^ Reports on the management of secondary TEF in critically ill patients are extremely rare due to the challenging nature of treatment and the absence of suitable therapeutic modalities.

The first case of successful closure of TEF with a septal occluder device was reported in 2010, involving a 58‐year‐old male patient who underwent surgical treatment for Barrett's esophageal adenocarcinoma.^16^ In that case, the septal occluder device achieved rapid closure of the leak, allowing for long‐term relief of respiratory symptoms. From a technical perspective, the device placement was straightforward, and no significant long‐term mucosal damage was observed during follow‐up. Based on these characteristics and advantages, it provided a direction for the more challenging and rescue treatment of TEF.

**FIGURE 3:**
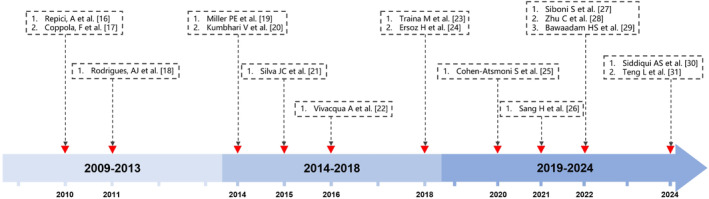
Timeline of tracheoesophageal fistula (TEF) treatment with Amplatzer Duct Occluder (ADO) and ADO‐like devices: A 15‐year literature review.

In our case, the patient's unstable condition and need for invasive mechanical ventilation precluded the use of SEMSs therapy. We chose the ADO II device under invasive mechanical ventilation to urgently occlude the patient's TEF, successfully preventing further deterioration of the patient's condition and allowing time for subsequent treatment. Based on respiratory and digestive endoscopic examinations and biopsy pathology results, it suggests that the cause of TEF is due to the recurrence of esophageal cancer. This may explain why the TEF did not heal after being occluded by ADO II. Although the ADO II did not achieve the purpose of curing the TEF, its successful placement effectively prevented further deterioration of the patient's condition, providing an opportunity for subsequent treatment. Figure 3 presents literature on the treatment of TEF using ADO or ADO‐like devices from the past 15 years, organized by publication date.^16^, ^17^, ^18^, ^19^, ^20^, ^21^, ^22^, ^23^, ^24^, ^25^, ^26^, ^27^, ^28^, ^29^, ^30^, ^31^

To the best of our knowledge, this is the first reported case of severe pneumonia with secondary TEF treated using sequential closure with an ADO II and a Y‐shaped tracheal stent during invasive mechanical ventilation. Selecting appropriate treatment modalities for the early and timely closure of secondary TEF is crucial for critically ill patients. ADO II can serve as a rescue therapy to achieve rapid closure of secondary TEF in critically ill patients requiring invasive mechanical ventilation support, providing a crucial window for further treatment.

## AUTHOR CONTRIBUTIONS

**Meng Fu:** Conceptualization; data curation; formal analysis; investigation; resources; software; writing – original draft; writing – review and editing. **Qixia Xu:** Conceptualization; supervision. **Jialiang Wang:** Software. **Dongsheng Wang:** Conceptualization; supervision. **Lejie Cao:** Conceptualization; supervision; writing – review and editing. **Junqiang Zhang:** Conceptualization; supervision; writing – review and editing.

## FUNDING INFORMATION

This study was funded by the Anhui Provincial Key Clinical Specialty Discipline Construction Program (2021szdzk05).

## CONFLICT OF INTEREST STATEMENT

The authors have no conflicts of interest to declare.

## ETHICS STATEMENT

All the treatment and study methods were carried out in accordance with the Declaration of Helsinki. Appropriate written informed consent was obtained for the publication of this case report and accompanying images. It was approved by the ethics committee of The First Affiliated Hospital of USTC (Ethical Approval Number: 2024‐RE‐62).

## CONSENT

Written informed consent was obtained from the patient to publish this report in accordance with the journal's patient consent policy.

## CONSENT FOR PUBLICATION

The publication of this case report and any accompanying images has obtained written informed consent from the patient's legal representative.

## Data Availability

All data and material are available for sharing if needed.
